# Supplementary material for: Mass Transport of Lignin in Confined Pores
Source: Polymers (Basel). 2022 May 13;14(10):1993. doi: 10.3390/polym14101993 (PMC9142885; doi:10.3390/polym14101993)
Supplement: Supplementary file 1 [file polymers-14-01993-s001.zip › polymers-1714074-supplementary.pdf]

Supplementary information

SI.1. Diffusion experiments – the water diffusion and water permeability over the membranes

According to the methodology previously described in the literature, the water permeability for all the regenerated cellulose membranes was measured using diffusion cells (1,2). Briefly, membranes were mounted between diffusion cells as described previously, 15 mL of deionized water was added to each of the donor and acceptor chambers simultaneously. 10 μL of 5 mCi (185 MBq) tritium-labelled water (PerkinElmer, USA) was added to the donor chamber and at specific time intervals, 500 μL samples were withdrawn from the acceptor chamber and replaced immediately with 500 μL of deionized water. Diffusion cells were placed on an orbital shaker (100 rpm) at room temperature. The withdrawn samples were analyzed by a liquid scintillation analyzer (Tri-Carb B2810TR, Perkin–Elmer, USA). To each sample, 3 mL of Ultima Gold solution (PerkinElmer, USA) was added prior to analysis. Experiments were conducted in triplicates. Water permeability in the membranes was calculated by equation 1, which based on a method developed by van den Mooter et al. 1994(3).

$$\frac{2PS}{hV}t = -\ln\left(\frac{C_0 - 2C_a}{C_0}\right) \qquad \text{Equation (S1)}$$

where D ($m^2\ s^{-1}$) is diffusivity, h (m) is the thickness of the membranes, S (m^2) is surface area of the membrane, V (m^3) is volume of solution, t (s) is time and C_0 and C_a (mol. m^3) are concentrations of donor at start and acceptor at time t, respectively. *Figure S1* shows the diffusivity values measured by radio diffusion method for all different membranes. For the small pore membranes, water diffusivity increased with size of the pores, however 25 kDa and 15 kDa are very close in water diffusivity.

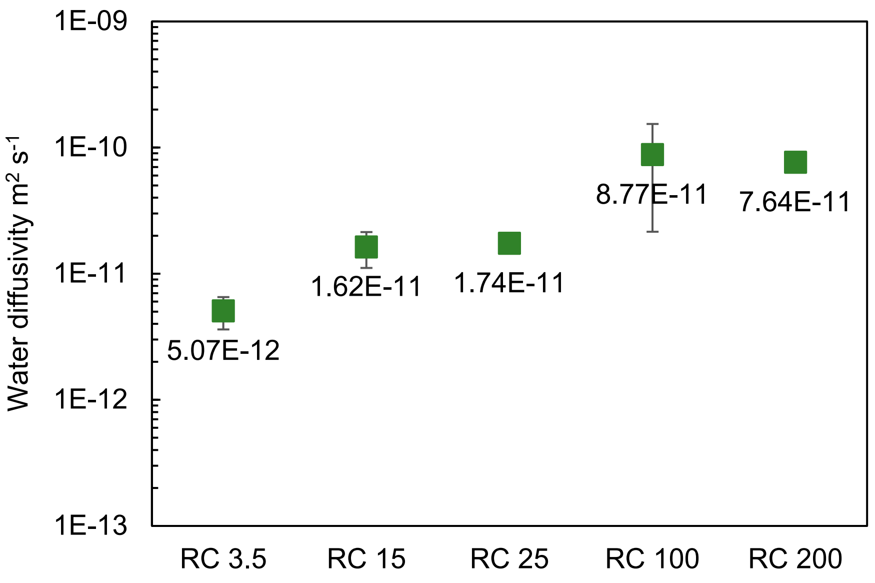

Figure S1. Water diffusivity of different membranes measured by diffusion of Tritium Oxide.

SI.2. SEM imaging of membranes

The used 100 nm and 200 nm membrane (RC100 and RC200 respectively) samples were gold-sputtered using a sputter coater (Q150 Plus Series, Quorum Technologies) for 60 seconds at 60 mA. The morphology of the sputtered samples was visualized using a scanning electron microscope (SEM) (JSM-7800FPRIME, JEOL) with an acceleration voltage of 5 kV.

Regenerated cellulose membranes with 100 nm (RC100) and 200 nm (RC200) nominal pore sizes were observed under the SEM after have being exposed to 0.1 M NaOH in the diffusion cells (*Figure S2*). Membranes after being used in diffusion cells do not show in breakage in fibers or in consistency in pores, therefore they are considered stable under the used conditions.

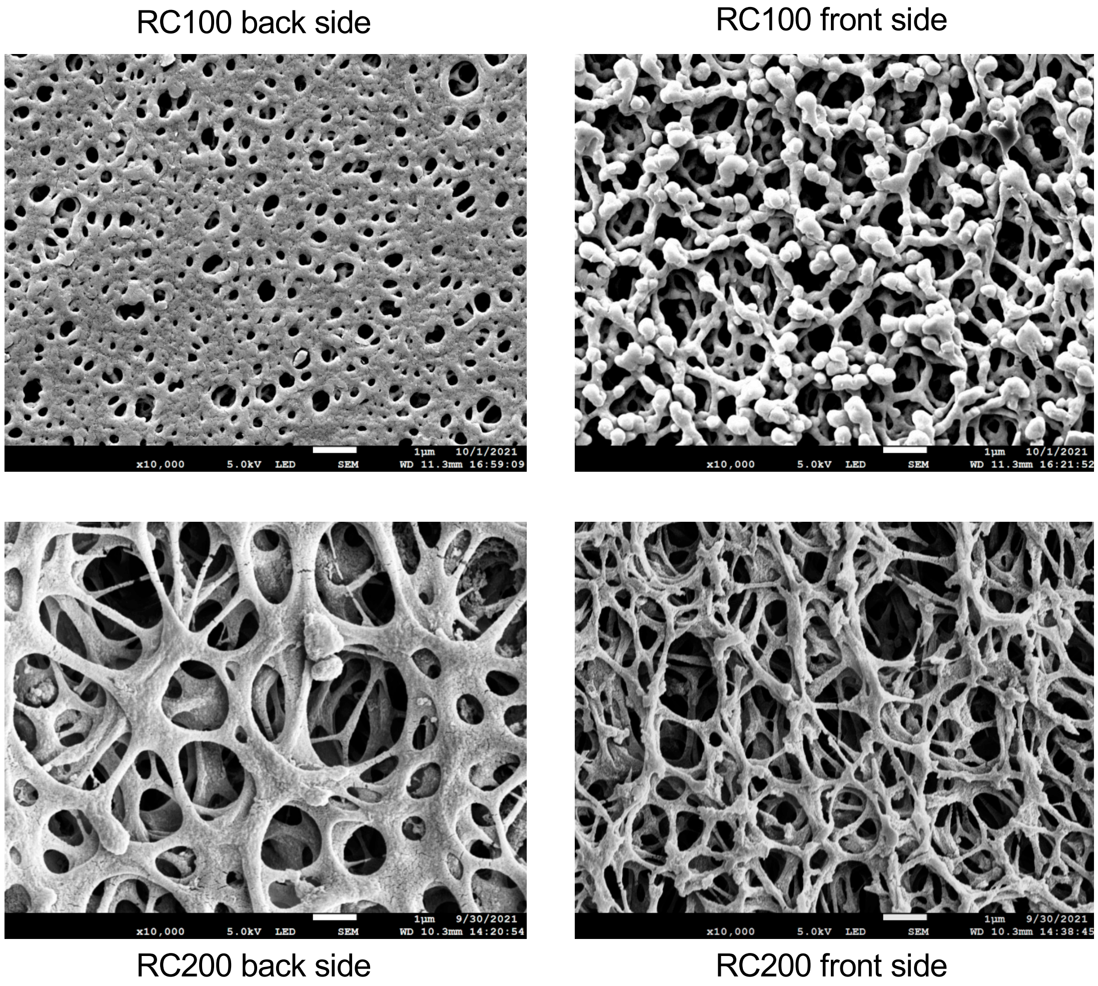

Figure S2. SEM images of used RC100 (top) and RC200 membranes (bottom).

SI.3. Molecular weight for the lignin molecules

Table S1 shows the summary of the average molecular weight of the lignin found in the acceptor side of each membrane after 168 hours since the start of the experiment. The M_w and Mn values presented here are based on the PEG standards used in the SEC system. While these standards are not the best representative for lignin molecules, the values here can be used for comparison among different samples. The KL molecules that passed through the small pores of RC3.5, RC15 and RC25 membranes are close in Mw, however there is a slight increase in M_w as the pore size of membranes increases. While all membranes have nominal pore sizes larger than the average M_w of KL in donor solution, only smaller molecules pass through RC3.5, RC15, and RC25. This shows that we can see a sort of molecular weight fractionation between donor and acceptor sides.

Table S1. Molecular weight values of KL species which were transported from donor through the membranes to the acceptor side. The absoulte molecular weight values here are based on PEG standards therefore these do not perfectly represent lignin molecules, it's best to use the values only for comparison.

	**Acceptor RC3.5**	**Acceptor RC15**	**Acceptor RC25**	**Acceptor RC100**	**Acceptor RC200**	**Donor KL in 0.1M NaOH**
**Mw (Da)**	953	1189	1458	3102	3077	3888
**Mn (Da)**	569	652	675	1098	1080	1393
**PDI**	1.67	1.82	2.16	2.82	2.85	2.79

SI.4. SEC Calibration curves and RI SEC chromatograms of acceptor samples

The RI chromatogram of PEG standards for calibration of SEC analysis is shown in Figure S3. As PEG does not absorb UV light RI signal is used to detect it. Kraft lignin on the other hand has too low signal on RI to be useful for detection in most cases (see Figure S4) therefore, we use the UV signals are used in SEC analysis.

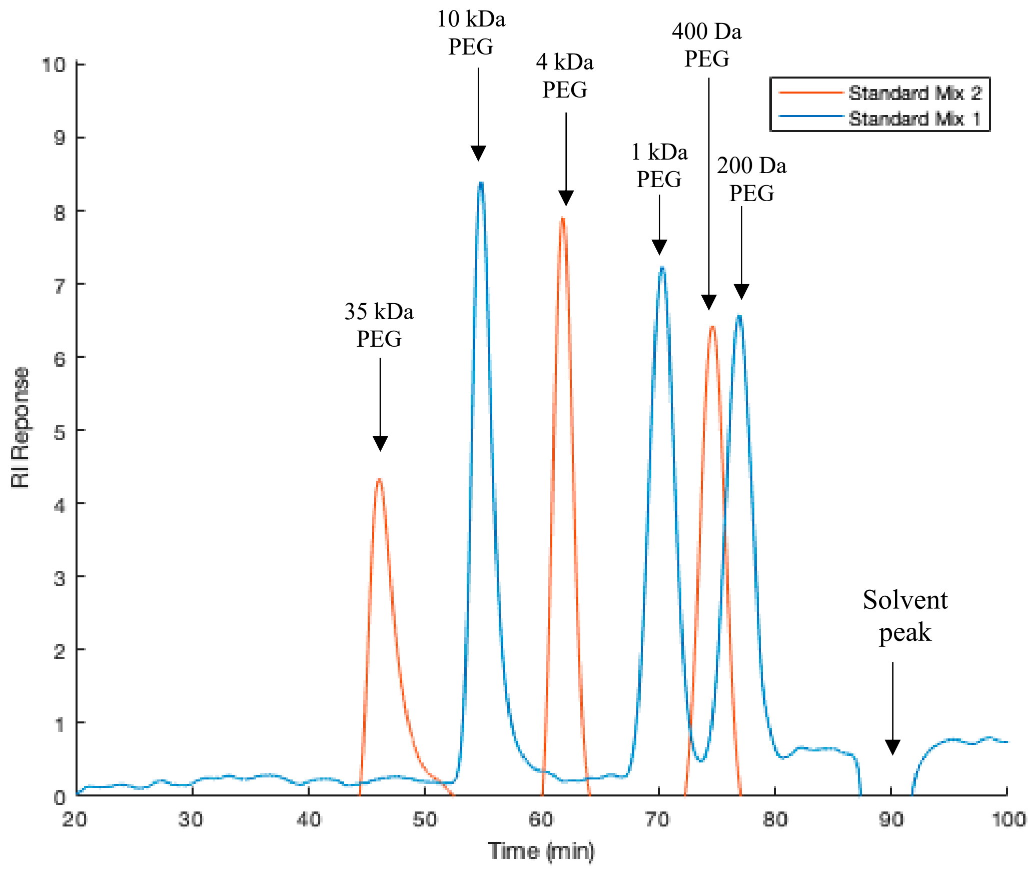

Figure S3. RI chromatogram of PEG standards for calibration of SEC analysis.

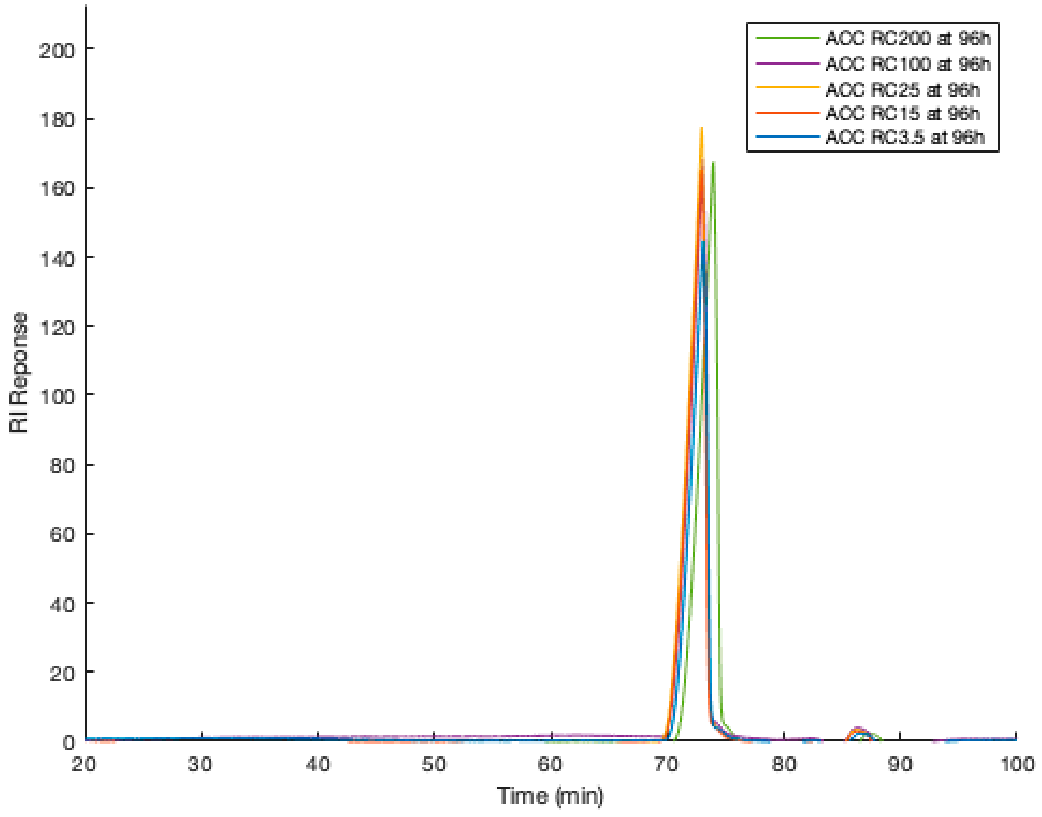

Figure S4. RI chromatogram of acceptor samples inf SEC analysis.

SI.5. Lignin conjugated structures

Some conjugated lignin structures can be found in Figure S5. Note that these are some examples of the conjugated structures which can show absorbance at 350 nm in UV spectrum, if dissolved in alkaline solutions (4,5). The real lignin structures can be larger molecules as well as the small ones which have these end units.

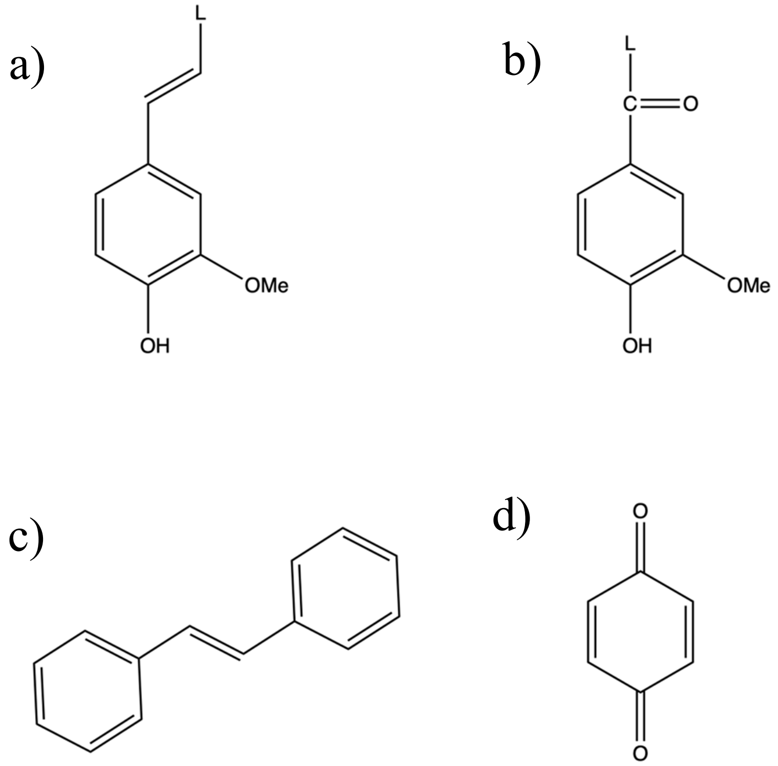

Figure S5. Types of phenolic conjugated structures in lignin.

References

1. Andersson H, Hjärtstam J, Stading M, Von Corswant C, Larsson A. Effects of molecular weight on permeability and microstructure of mixed ethyl-hydroxypropyl-cellulose films. Eur J Pharm Sci [Internet]. 2013;48(1–2):240–8. Available from: http://dx.doi.org/10.1016/j.ejps.2012.11.003
2. Nilsson R, Olsson M, Westman G, Matic A, Larsson A. Screening of hydrogen bonds in modified cellulose acetates with alkyl chain substitutions. Carbohydr Polym [Internet]. 2022;285(November 2021):119188. Available from: https://doi.org/10.1016/j.carbpol.2022.119188
3. Van den Mooter G, Samyn C, Kinget R. Characterization of colon-specific azo polymers: A study of the swelling propertoes and the permeability of isolated polymer films. Int J Pharm. 1994;111(2):127–36.
4. Gärtner A, Gellerstedt G, Tamminen T. Determination of phenolic hydroxyl groups in residual lignin using a modified UV-method. Nord Pulp Pap Res J. 1999;14(2):163–70.
5. Bikova T, Treimanis A, Rossinska G, Telysheva G. On-line study of lignin behaviour

in dilute alkaline solution by the SEC-UV method. Holzforschung. 2004;58(5):489–94.
